# dOFV distributions: a new diagnostic for the adequacy of parameter uncertainty in nonlinear mixed-effects models applied to the bootstrap

**DOI:** 10.1007/s10928-016-9496-7

**Published:** 2016-10-11

**Authors:** Anne-Gaëlle Dosne, Ronald Niebecker, Mats O. Karlsson

**Affiliations:** Department of Pharmaceutical Biosciences, Uppsala University, P.O. Box 591, 751 24 Uppsala, Sweden

**Keywords:** Parameter uncertainty distributions, Bootstrap, Model diagnostics, Nonlinear mixed-effects models

## Abstract

Knowledge of the uncertainty in model parameters is essential for decision-making in drug development. Contrarily to other aspects of nonlinear mixed effects models (NLMEM), scrutiny towards assumptions around parameter uncertainty is low, and no diagnostic exists to judge whether the estimated uncertainty is appropriate. This work aims at introducing a diagnostic capable of assessing the appropriateness of a given parameter uncertainty distribution. The new diagnostic was applied to case bootstrap examples in order to investigate for which dataset sizes case bootstrap is appropriate for NLMEM. The proposed diagnostic is a plot comparing the distribution of differences in objective function values (dOFV) of the proposed uncertainty distribution to a theoretical Chi square distribution with degrees of freedom equal to the number of estimated model parameters. The uncertainty distribution was deemed appropriate if its dOFV distribution was overlaid with or below the theoretical distribution. The diagnostic was applied to the bootstrap of two real data and two simulated data examples, featuring pharmacokinetic and pharmacodynamic models and datasets of 20–200 individuals with between 2 and 5 observations on average per individual. In the real data examples, the diagnostic indicated that case bootstrap was unsuitable for NLMEM analyses with around 70 individuals. A measure of parameter-specific “effective” sample size was proposed as a potentially better indicator of bootstrap adequacy than overall sample size. In the simulation examples, bootstrap confidence intervals were shown to underestimate inter-individual variability at low sample sizes. The proposed diagnostic proved a relevant tool for assessing the appropriateness of a given parameter uncertainty distribution and as such it should be routinely used.

## Introduction

The utilization of non-linear mixed effects models (NLMEM) in drug discovery and development has increased in the last decades. In a recent white paper aimed at both decision makers and practitioners, the importance and implementation of Model-Informed Drug Discovery and Development (MID3) was outlined [1]. As exemplified and stressed in this consensus paper, understanding and quantifying model uncertainty has a central role in model-informed decision-making. Model uncertainty includes qualitative aspects, mainly the adequacy of the assumptions underpinning the model(s), but also quantitative aspects, mainly the joint uncertainty of the estimated parameters of the model(s). A number of methods to quantify parameter uncertainty are available. The most commonly used method is to derive standard errors around the parameters from the asymptotic covariance matrix, which is provided as a standard output of most software. A drawback of this uncertainty estimate is that parameter confidence intervals (CI) as well as simulation and prediction uncertainties require additional assumptions about the shape of the uncertainty distribution. Most often a multivariate normal distribution is used, which provides CI that are symmetric around the point estimate. To relax the assumption of symmetric CI, likelihood profiling is sometimes used [2]. However, while applicable to the uncertainty of each parameter separately, likelihood profiling does not allow the use of the combined uncertainty around all model parameters for simulations or predictions. The most frequently used method for generation of parameter uncertainty without symmetry constraint is the nonparametric, or “case”, bootstrap [3]. Case bootstrap of independent data consists of estimating model parameters on a number of datasets obtained by resampling with replacement “pairs” of dependent and independent variables. It is often considered as the gold standard for estimating parameter uncertainty. Occasionally, other bootstrap variants are used, for example nonparametric and parametric residual bootstrap [4]. Sampling Importance Resampling (SIR) has recently been proposed for improving the estimation of parameter uncertainty in NLMEM [5]. Lastly, for models estimated by Bayesian methods, the full posterior parameter distribution is typically used to represent parameter uncertainty [6].

 Case bootstrap is not devoid of limitations. It is for example not suitable for “small” or highly designed datasets for which stratification is impossible (such as model-based meta-analysis [7]) or leads to too small subgroups. Using bootstrap in these cases has a high risk of leading to biased uncertainty estimates. Models relying on frequentist parameter priors for parameter estimation [8] are also not suited for bootstrap, as the uncertainty of parameters featuring highly informative priors and low information in the data will be underestimated. In the particular context of NLMEM, what represents a too small dataset is not well known. In any case, a minimum dataset size would need to take model complexity into account. As models typically grow in complexity with increasing dataset sizes in order to maximize the amount of information extracted from the data, one might suggest that datasets are often small with regards to the developed models. In addition, typically each individual, or “case”, only provides incomplete information about the parameters that are to be estimated. Likewise, individuals contain different amounts of information and are thus not fully exchangeable. Reasons for this may be different designs (doses, number or timing of observations) and different covariate values, but even with the same design and covariate values, the information about a parameter will be linked to the individual’s value for that parameter and thus differ between “cases”.

 The appropriateness of the point estimates of model parameters is typically scrutinized using a variety of graphical diagnostics based on predictions, residuals and simulations. The choice of models and estimation methods in an analysis is typically driven by such diagnostics. The same is not true for estimates of parameter uncertainty. Typically, the appropriateness of the uncertainty estimates is not investigated. Instead, the choice of method for the estimation of parameter uncertainty is usually based on the expectation of performance combined with practical aspects (runtime, model stability, etc.). In the present work, we propose a diagnostic for estimates of parameter uncertainty. The intention for its use is mainly to allow assessment of the appropriateness of estimated parameter uncertainty in relation to the underlying real dataset, but its properties will also be explored in relation to simulated data. For illustration of the parameter uncertainty diagnostic we have chosen to apply it to case bootstrap estimates.

## Methods

### NLMEM

A NLMEM is a statistical model which typically describes how an endpoint changes over time in different individuals. It is usually defined by a set of differential equations and comprises *N*_*par*_ population parameters. The vector of population parameters *P* comprises both fixed and random effects, which can be combined to obtain vectors of individual parameters *P*_*i*_. Estimates of the population parameters, $\hat{P}$, can be obtained from a dataset *D* containing a total number of observations *N*_*obs*_ arising from *N*_*id*_ individuals by minimization of the objective function value (OFV), which is equal (up to a constant) to minus two times the log-likelihood of the data given the parameters.

### Bootstrap setup

Uncertainty in the population parameters estimated on dataset *D* can be obtained via bootstrap. Bootstrap results considered in this work consisted of *N*_*boot*_ = 1000 parameter vectors obtained by fitting the model, i.e. estimating the population parameters, in *N*_*boot*_ bootstrapped datasets. The bootstrapped datasets were obtained from the original data using case bootstrap, where the full data of one individual was resampled with replacement to obtain bootstrapped datasets containing the same number of individuals as the original dataset. Some individuals are thus present multiple times within a bootstrapped dataset, in which case they are treated as independent individuals; oppositely, some individuals are not present at all. Stratification, i.e. the classification of individuals into subgroups prior to resampling, was not used here. Model fitting of the bootstrapped datasets was performed the same way as with the original dataset, with initial parameter estimates set to $\hat{P}$. Nonparametric percentiles-based CI were derived for each parameter from all available bootstrap parameter vectors, regardless of their termination status (i.e. runs with and without successful minimization were used to compute bootstrap CI).

### dOFV distribution diagnostic

A diagnostic was developed to assess whether the uncertainty of NLMEM parameter estimates, as obtained by the bootstrap procedure described above, was appropriate.

The new diagnostic is based on the comparison of the distribution of the differences in OFV (dOFV) for a proposed uncertainty estimate, in this case the bootstrap, to a theoretical dOFV distribution. The two dOFV distributions are generated according to the following procedure:

#### Bootstrap dOFV distribution

The bootstrap dOFV distribution is derived from the results of the bootstrap of the original dataset, which consist of *N*_*boot*_ parameter vectors. Each one of the *N*_*boot*_ parameter vectors has been estimated on a different bootstrapped dataset. Each of these vectors is then used to evaluate the likelihood of the original data given this particular vector. For one vector, this means that the OFV of the original data is calculated by fixing all model parameters to their value in the vector (in the NONMEM [13] software, this corresponds to using the bootstrap parameter vector as initial estimates in $THETA, $OMEGA and $SIGMA and setting MAXEVAL = 0 in $EST). This is done for each of the *N*_*boot*_ vectors, leading to *N*_*boot*_ OFVs. Next, the final OFV of the original model, obtained with the final parameter estimates on the original data (using MAXEVAL = 9999 in $EST), is subtracted from each of the *N*_*boot*_ OFVs. One thus obtains *N*_*boot*_ dOFVs (Eq. 1).

1 $$dOFV_{bootN} \; = \;OFV_{{\hat{P}_{bootN} , D}} \; - \;OFV_{{\hat{P}_{D} , D}}$$

where $dOFV_{bootN}$ is the *N*-th bootstrap dOFV. The first index of the OFV corresponds to the parameter vector used, and the second to the dataset the parameter vector is estimated (MAXEVAL = 9999 in NONMEM) or evaluated (MAXEVAL = 0) on. $\hat{P}_{bootN}$ is the parameter vector estimated on the *N*-th bootstrap dataset, and $\hat{P}_{D}$ is the parameter vector estimated on the original dataset *D.*

#### Theoretical dOFV distribution

The second dOFV distribution, referred to as the theoretical dOFV distribution, corresponds to a Chi square distribution with degrees of freedom (df) equal to the number of estimated model parameters *N*_*par*_ (Eq. 2).

2 $$dOFV_{theoreticalN} \; = \;random(\chi_{Npar}^{2} )$$

where $dOFV_{theoreticalN}$ is the *N*-th Chi square dOFV obtained by random sampling from a Chi square distribution with $N_{par}$ degrees of freedom.

**Fig. 1:**
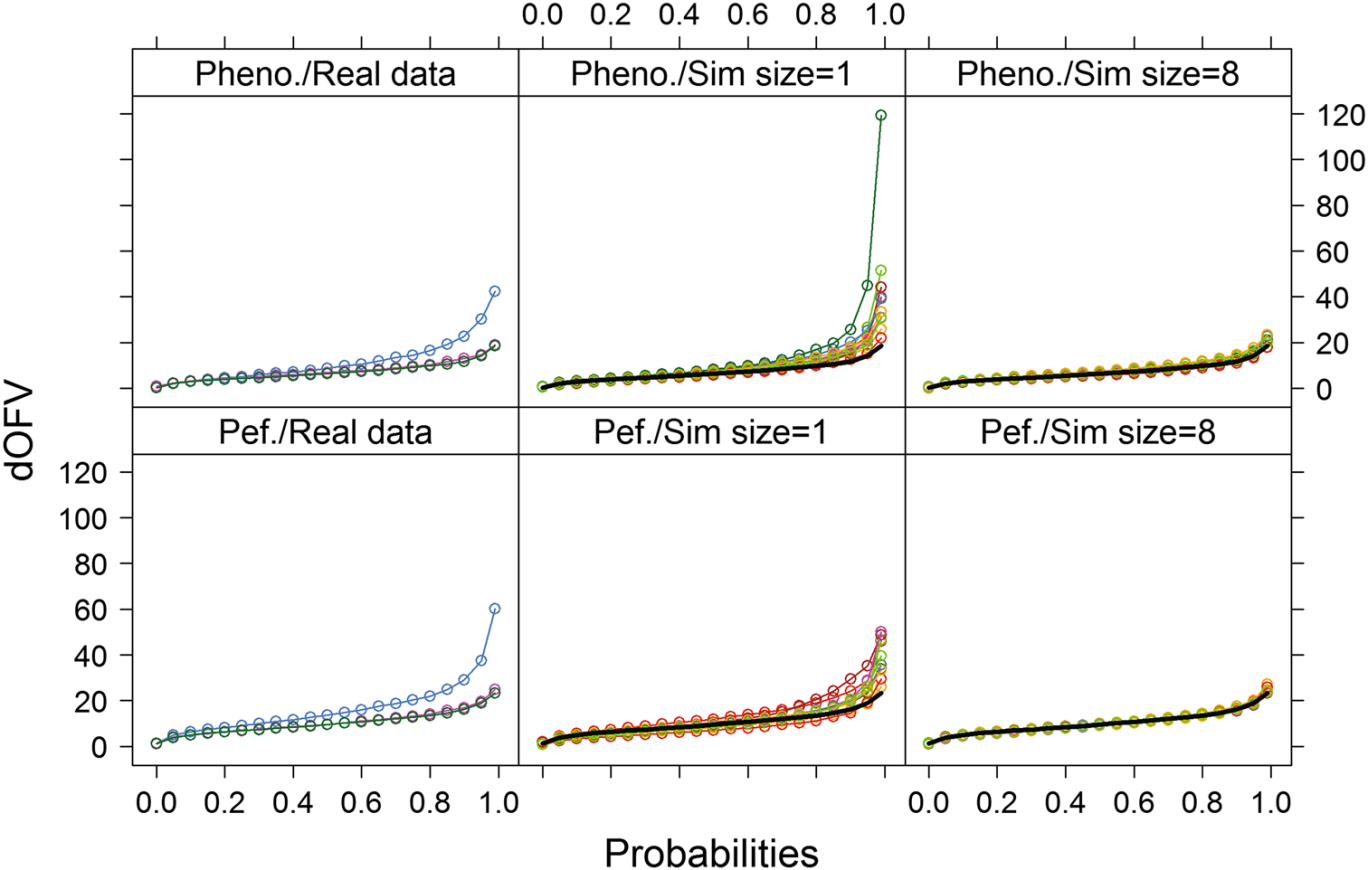
dOFV distribution plots for the two real data examples. *Left panels* provide bootstrap dOFV distributions for the real data (*blue*), the theoretical dOFV distribution (*green*) and the SSE dOFV distribution (*pink*). *Middle* and *right panels* provide bootstrap dOFV distributions for the simulated datasets of equal and 8-fold increased size (*colors*), as well as the theoretical dOFV distribution (*black solid line*). *Pheno.* phenobarbital, *Pef.* pefloxacin

The proposed diagnostic displays the quantile functions, also known as the inverse cumulative distribution functions, of the two dOFV distributions as illustrated in Fig. 1 (left panels—the bootstrap dOFV distribution is displayed in blue and the theoretical dOFV distribution is displayed in green). The quantile function specifies, for a given probability in the probability distribution of a random variable, the value at which the probability of the random variable being less than or equal to this value is equal to the given probability.

The principle behind the dOFV diagnostic is that if the parameter vectors (obtained by performing a bootstrap for example) were representative of the true uncertainty, their dOFV distribution should follow a Chi square distribution with a certain degrees of freedom. For unconstrained fixed effects models, asymptotically the degrees of freedom of the Chi square distribution should be equal to the number of estimated parameters [9], and thus the bootstrap dOFV distribution should overlay the theoretical dOFV distribution. However for NLMEM the exact degrees of freedom is unknown. It could be equal or inferior to the number of estimated parameters, notably due to the estimation of random effects and other bounded parameters, which may not account for full degrees of freedom. Other factors influencing the dOFV distribution may be properties of the estimation method (e.g. first or second-order approximations of the likelihood [10]) and the presence of model misspecification. Because the true degrees of freedom is unknown, the bootstrap dOFV distribution is not necessarily expected to collapse to the theoretical dOFV distribution when the uncertainty is appropriate. In the absence of model misspecification, it is however expected to collapse to the dOFV distribution obtained by stochastic simulations and estimations (SSE, as described below). The SSE dOFV distribution takes all model properties such as the presence of random effects, boundaries, or the estimation method into account, and thus represents the expected dOFV distribution in the absence of model misspecification. The SSE dOFV distribution was computed for the purpose of evaluating the proposed diagnostic, but it is important to note that it is not part of the diagnostic in practice due to its high computational burden. Instead, it is computed here to evaluate whether the theoretical distribution is a good enough surrogate for the SSE dOFV distribution for the investigated NLMEM. The SSE dOFV distribution is obtained as follows:

#### SSE dOFV distribution

*N*_*SSE*_ = 1000 datasets are simulated based on the given model, with simulation parameters equal to the parameters $$\hat{P}_{D}$$ estimated on the original data. For each simulated dataset, two OFVs are computed: the OFV obtained by estimating model parameters on the simulated dataset (using the simulation parameters as initial estimates in $THETA, $OMEGA and $SIGMA and setting MAXEVAL = 9999 in $EST), and the OFV obtained by evaluating the simulation parameters on the simulated dataset (using the simulation parameters as initial estimates in $THETA, $OMEGA and $SIGMA and setting MAXEVAL = 0 in $EST). A dOFV is then computed for each dataset as the difference between the second and the first OFV. One thus obtains *N*_*SSE*_ SSE dOFVs (Eq. 3).

3 $$dOFV_{SSEN} \; = \;OFV_{{\hat{P}_{SSEN} , SSEN}} \; - \;OFV_{{\hat{P}_{D} , SSEN}}$$

$dOFV_{SSEN}$ is the *N*-th SSE dOFV. The first index of the OFV corresponds to the parameter vector used, and the second to the dataset the parameter vector is estimated (MAXEVAL = 9999 in NONMEM) or evaluated (MAXEVAL = 0) on. $\hat{P}_{SSEN}$ is the parameter vector estimated on the *N*-th SSE dataset, and $\hat{P}_{D}$ is the parameter vector estimated on the original dataset *D*.

The dOFV diagnostic assesses whether a given uncertainty method is appropriate for a given dataset and model: the uncertainty is considered appropriate if its dOFV distribution is at or below the theoretical distribution. In addition, in this work we evaluated whether using the theoretical dOFV distribution as a surrogate for the SSE distribution was appropriate.

The adequacy of parameter uncertainty obtained by bootstrap was evaluated based on two real data examples and two simulation examples using the new dOFV distribution diagnostic as well as other parameter distribution metrics.

For the real data examples, bootstrap dOFV distributions were assessed for (i) the original dataset, (ii) 10 datasets simulated with the final model and parameters estimates using the original design, and (iii) 10 datasets simulated with the final model and parameters estimates using the original design but with an 8-fold increase in the number of individuals. From these investigations both the influence of dataset size (original size for i and ii, increased size for iii) and model misspecification (potential misspecification for i, no misspecification for ii and iii) on bootstrap uncertainty adequacy could be assessed. SSE and theoretical dOFV distributions were assessed for all scenarios. Note that SSE dOFV distributions are identical for i and ii, and that theoretical dOFV distributions are identical for i, ii and iii.

For the simulation examples, bootstrap dOFV distributions were assessed for 100 simulated datasets for each dataset size. SSE and theoretical dOFV distributions were also assessed. The adequacy of parameter uncertainty was further evaluated based on parameter CI, using parameter CI obtained from the SSE (using 1000 samples) as the reference. Coverage at the 90 % level was investigated for each parameter by calculating the percentage of datasets for which the 90 % CI included the true simulation value for that parameter.

### Investigated examples

Table 1 provides a summary of all investigated examples.

**Table 1:** Summary of the investigated real data and simulation examples

Example	Model	Total number of parameters	Number of random effect parameters (proportion)	Number of individuals	Number of observations	Number of observations/individual
Phenobarbital [11]	1-compartment PK model, multiple i.v. doses, linear elimination	7	3 (0.43)	59, 472^a^	155, 1240^a^	2.6
Pefloxacin [12]	1-compartment PK model, multiple i.v. doses, linear elimination	10	7 (0.70)	74, 592^a^	337, 2696^a^	4.6
Simulation 1 (PK)	1-compartment PK model, single i.v. dose, linear elimination	5	3 (0.60)	20, 50, 200	80, 200, 800	4
Simulation 2 (PD)	Emax PD model with baseline	7	3 (0.43)	20, 50, 200	80, 200, 800	4

*PK* pharmacokinetic, *PD* pharmacodynamic, *i.v.* intravenous

^a^Simulated data (8-fold increase in the number of individuals compared to the original dataset)

#### Phenobarbital

The phenobarbital dataset [11] consisted of 155 observations from 59 infants. Phenobarbital pharmacokinetics (PK) were described by a one-compartment model with multiple i.v. bolus administration and linear elimination. The model included seven parameters: clearance (CL) and volume of distribution (V) with inter-individual variability (IIV), two covariate relationships (body weight on CL and V), and additive residual unexplained variability (RUV).

#### Pefloxacin

The pefloxacin dataset [12] consisted of 337 observations from 74 critically ill patients. The PK of pefloxacin was described by a one-compartment model with multiple i.v. bolus administration and linear elimination. The model included ten parameters: CL and V with IIV and inter-occasion variability (IOV) with correlations between the variabilities of CL and V within a level, one covariate relationship (creatinine clearance on CL), and proportional RUV.

#### Simulation 1

Data was simulated from a one-compartment PK model with a single i.v. bolus administration of 100 and linear elimination. The model included five parameters: CL and V, equal to 1 and each displaying 30 % exponential IIV, and an additive RUV on the log scale with a standard deviation of 0.2. Three different dataset sizes were investigated: 20, 50 and 200 individuals, with four observations each at 0.25, 0.5, 1 and 2 units post dose.

#### Simulation 2

Data was simulated from a pharmacodynamic (PD) dose–response sigmoidal Emax model with baseline. The model included seven parameters: a baseline (E0) of 10, an additive maximum effect (EMAX) of 100 with a dose leading to half the maximum effect (ED50) of 5, a Hill factor (HILL) of 0.7, 30 % exponential IIV on E0 and ED50, and a proportional RUV with a standard deviation of 10 %. Three different dataset sizes were investigated: 20, 50 and 200 individuals with four observations each at doses of 0, 2.5, 5 and 15.

### Software

Data simulation and analysis including parameter estimation and evaluation was performed with NONMEM 7.2 [13] using PsN [14] as a modelling environment. The dOFV diagnostic is fully automated for bootstrap using the -dOFV option in PsN. Post-processing and graphical output was performed in Rstudio 0.99.484 with R 3.2.1 [15].

## Results

### Real data examples

In the two investigated real data examples, theoretical and SSE dOFV distributions were almost superimposed whichever dataset size (Fig. 1). Bootstrap dOFV distributions showed different patterns for different dataset types (observed vs. simulated data) and sizes.

For the original datasets (left panel of Fig. 1), bootstrap dOFV distributions deviated clearly from the theoretical dOFV distributions, with dOFVs consistently higher than expected from the theoretical distribution. Estimated degrees of freedom were 4–7 df (i.e. around 65 %) higher than their reference, at 11.4 and 16.5 instead of 7 and 10 for phenobarbital and pefloxacin, respectively (Table 2).

**Table 2:** Degrees of freedom of the dOFV distributions for the real data and simulation examples

Real data examples	Df Chi square (theoretical)	Df original	Df sim 1× median (range)	Df sim 8× median (range)
Phenobarbital [11]	7	11.4	8.81 (6.86, 14.3)	7.35 (6.45, 8.45)
Pefloxacin [12]	10	16.5	11.3 (8.48, 14.0)	10.0 (9.78, 10.9)

Simulation examples	Df Chi square (theoretical)	Df 20-4 median (range)	Df 50-4 median (range)	Df 200-4 median (range)
Simulation 1 (PK)	5	6.25 (4.32, 10.6)	5.48 (4.10, 7.15)	5.07 (4.42, 5.94)
Simulation 2 (PD)	7	8.39 (5.74, 14.4)	7.55 (6.22, 9.93)	7.15 (6.24, 8.08)

For the simulated datasets of equal size (middle panel of Fig. 1), bootstrap dOFV distributions also deviated from the theoretical dOFV distributions, although the extent of deviation was reduced. The median degrees of freedom was 8.8 instead of 7 for phenobarbital (i.e. around 26 % higher) and 11.3 instead of 10 for pefloxacin (i.e. around 13 % higher). Differences in degrees of freedom between datasets were high, with degrees of freedom spanning 5.5–7.5 df.

For the simulated datasets of eight-time increased size (right panel of Fig. 1), bootstrap dOFV distributions were much closer to the theoretical dOFV distributions: the median degrees of freedom was 7.4 instead of 7 for phenobarbital (i.e. around 6 % higher) and exactly 10 for pefloxacin (i.e. no increase). Differences in degrees of freedom between datasets were less marked than with datasets of equal size, with degrees of freedom spanning 1–2 df.

### Simulation examples

**Fig. 2:**
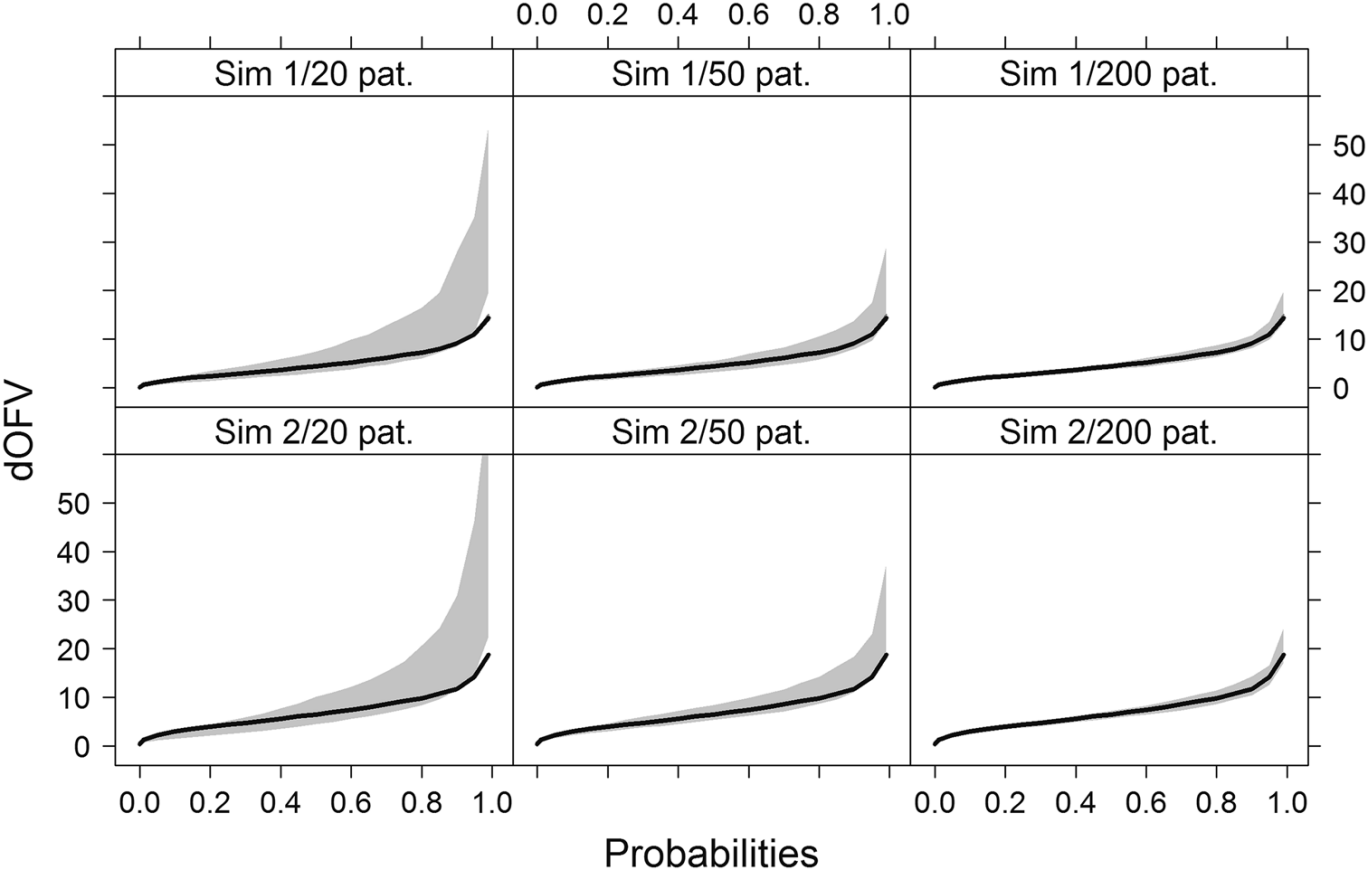
dOFV distribution plots for the two simulation examples. *Grey shaded areas* represent the range of dOFV curves for n = 100 bootstraps, with the theoretical dOFV distribution superimposed (*solid black line*). One panel corresponds to one simulation example and dataset size. *Sim* simulation, *pat.* patients

As for the real data examples, theoretical and SSE dOFV distributions were almost superimposed whichever dataset size (Fig. 2), and bootstrap dOFV distributions showed different patterns for different dataset sizes. Bootstrap dOFV distributions behaved similarly for the two simulation examples.

The range of bootstrap dOFV distributions decreased with increasing dataset size, and approached more and more the theoretical dOFV distribution. For the smallest dataset size of 20 patients (left panel of Fig. 2), the mean bootstrap degrees of freedom was 25 % higher than the theoretical for the PK example and 20 % higher for the PD example (Table 2). With 50 patients (middle panel of Fig. 2), discrepancies were reduced to 10 % increases over the theoretical for both examples. At the highest sample size of 200 patients (right panel of Fig. 2), discrepancies had almost disappeared. As previously observed with the real data examples, differences in degrees of freedom between datasets also decreased with increasing dataset size. Ranges decreased from 6 to 1.5 df in the PK example and from 8 to 2 df in the PD example, approximately halving at each dataset size.

**Fig. 3:**
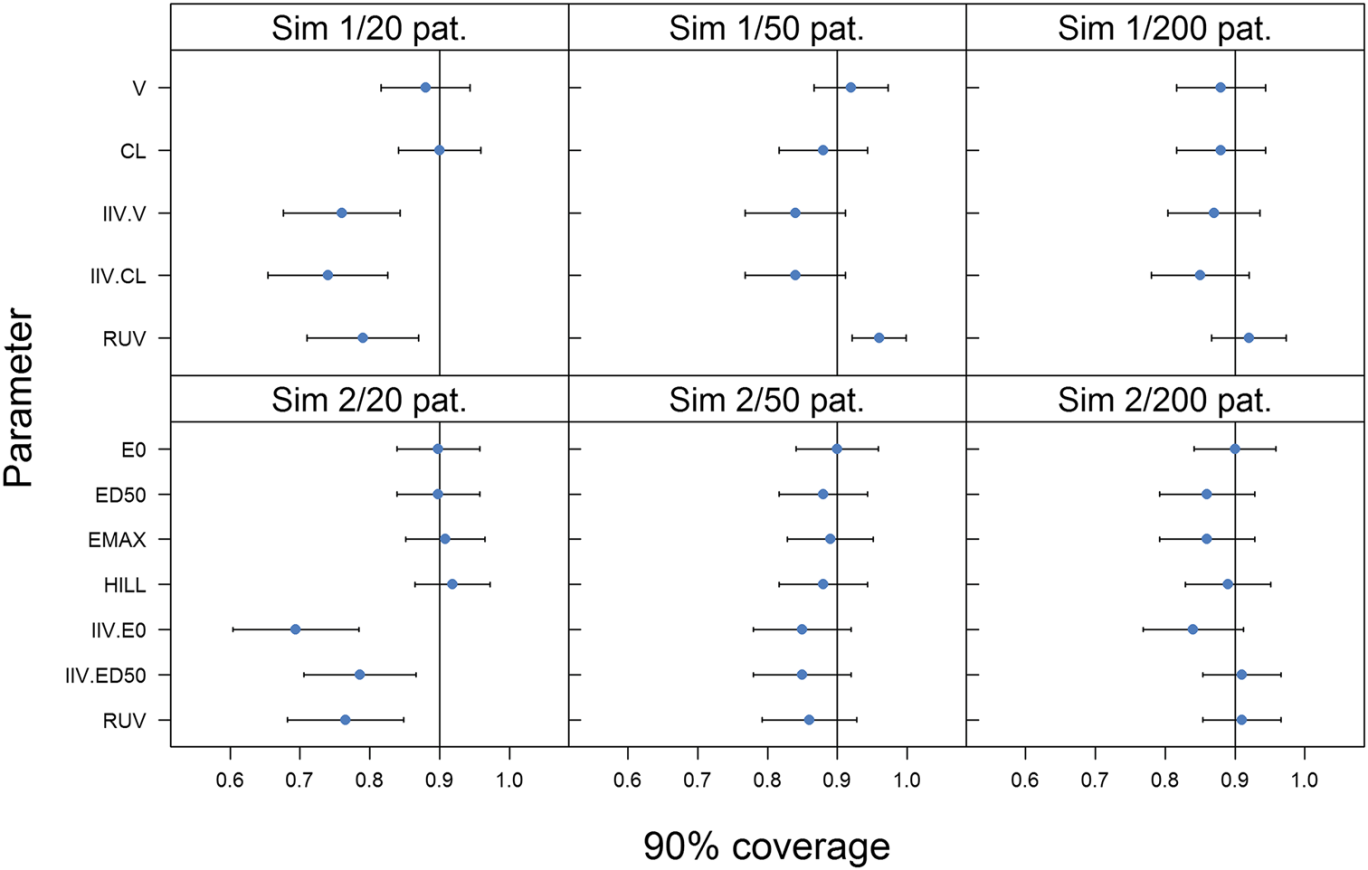
90 % coverage for all parameters of the two simulation examples. One panel corresponds to one simulation example and dataset size. *CL* clearance, *V* volume of distribution, *IIV* inter-individual variability, *RUV* residual unexplained variability, *E0* baseline, *EMAX* maximum effect, *ED50* dose leading to half the maximum effect, *HILL* Hill factor, *Sim* simulation, *pat*. patients

For the simulation examples, further properties of the bootstrap could be investigated as the true uncertainty distribution could be estimated from the simulations. Figure 3 displays the 90 % coverage of model parameters for each dataset size. Trends with increasing dataset size were similar between the two examples: bootstrap coverage was always satisfactory for fixed effects, but deviations from the expected coverage were observed for random effects at the lowest sample size and to a lesser extent at the middle sample size. Coverage of IIV and RUV were between 0.70 and 0.80 instead of 0.90 for datasets with 20 patients in both examples. With 50 patients, coverage increased to 0.85 except for the RUV of the PK example, which peaked at 0.95. Coverage of all random effects was close to the expected level with 200 patients.

**Fig. 4:**
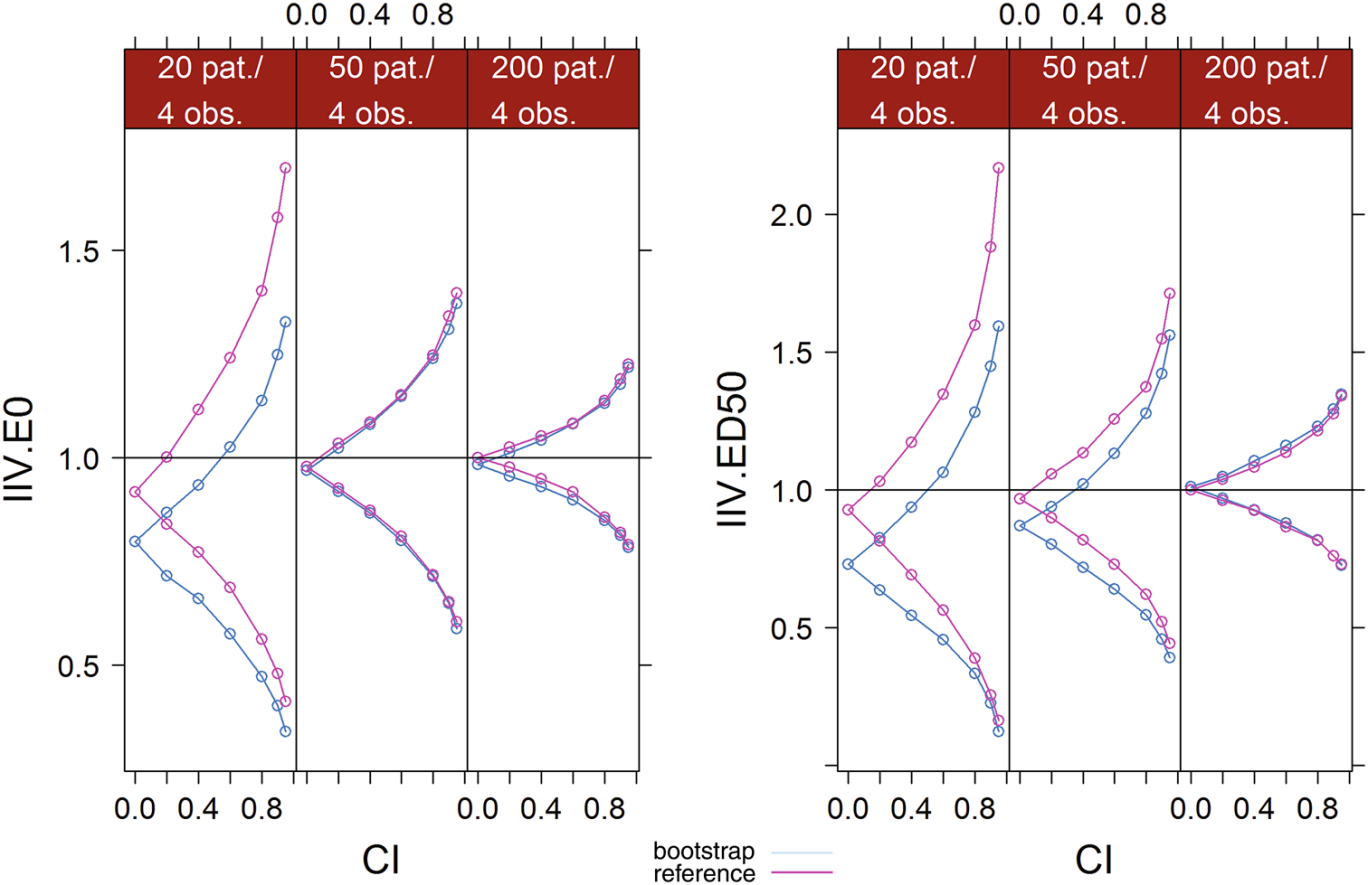
Comparative CI bounds between the bootstrap and the reference (SSE) at different confidence levels for the IIV parameters of the second simulation example. Values were normalized by the true simulation value. For the bootstrap, median values over all simulation are displayed. *IIV* inter-individual variability, *E0* baseline, *EMAX* maximum effect, *ED50* dose leading to half the maximum effect, *CI* confidence level, *pat.* patients, *obs.* observations

Figure 4 displays the outer bounds of increasing CI levels of the IIV parameters of the PD example based on the bootstrap and on the reference SSE for each dataset size. Values were normalized by the true simulation value of each parameter. Discrepancies between the bootstrap and the reference CI bounds were apparent at low sample sizes, but disappeared as sample size increased to 50 patients for IIV E0 or to 200 patients for IIV ED50. With 20 patients, bootstrap underestimated the uncertainty of all IIV parameters. Both bounds were underestimated, meaning that the CI were not only too narrow but also shifted down. Medians of the bootstrap distributions (i.e. CI = 0) were 20–25 % lower than the true simulation parameter. Some downward bias was also observed for the medians of the SSE. Bootstrap upper bounds were consistently below the reference bounds, with differences increasing with increasing CI: the upper bound of the 95 % CI of IIV E0 was 35 % above the simulation value with bootstrap, versus 70 % with the reference. For IIV ED50, this value was 60 % with bootstrap instead of 120 % with the reference. Lower bounds were also below the reference bounds, but differences decreased with increasing CI and were less marked. For IIV E0, the lower bound of the 95 % CI was 60 % below the simulation value with bootstrap, versus 55 % with the reference. For IIV E0, the lower bound of the bootstrap 90 % CI converged to the lower bound of the reference, 80 % below the simulation value. With 50 patients, bootstrap and reference CI bounds for IIV E0 overlapped at all CI and medians were at the true simulation value. This was not the case for IIV ED50, which displayed similar patterns with 50 patients than with 20 patients, even if differences between the bootstrap and the reference were less marked. With 200 patients, bootstrap and reference CI bounds overlapped for all CI for both IIV.

## Discussion

The dOFV diagnostic enabled to assess whether a given uncertainty estimate could be considered adequate. When it was not, i.e. when the dOFV distribution of the bootstrap was above the theoretical distribution, it translated into suboptimal coverage of the random effects, for which both the medians and the CI widths were underestimated. Difference of about 1.5 df led to an underestimation of the 90 % coverage by 5–20 %, and to underestimations of medians and 90 % CI widths by about 20 %. Diagnosing a priori in which cases bootstrap is inadequate based on sample size proved inappropriate for NLMEM, as will be discussed below. An a posteriori method, based on parameter-specific “effective” sample sizes, will be proposed. In the investigated examples, effective sample sizes greater than 45 individuals led to good coverage and CI, and could be used to identify parameters which uncertainty was not well described by the bootstrap. Depending on the purpose of the modelling, this could for example be addressed by using a different uncertainty method less sensitive to sample size, such as SIR [5].

### Development and use of the dOFV distribution diagnostic

To the authors’ knowledge, this is the only currently available diagnostic of this kind for NLMEM, and potentially for other types of models as well. The dOFV diagnostic is a global test: as the dOFV distributions do not differentiate between parameters, it does not indicate for which parameter(s) the uncertainty is not well described. The diagnostic was exemplified on bootstrap, but it can be applied to any method for assessing parameter uncertainty, provided parameter vectors can be drawn from the proposed uncertainty distribution (which is not the case for likelihood profiling for example). The developed diagnostic is based on evaluating the likelihood of the data at hand for a number of parameter vectors drawn from the proposed uncertainty. If the proposed uncertainty is the true uncertainty, differences between these likelihoods and the likelihood of the final parameter estimates, summarized by the dOFV distribution, are expected to follow a Chi square distribution. Given the importance of parameter uncertainty in decision-making, there is no reason why scrutiny towards uncertainty estimates should be ignored. Along with other efforts towards a standardization of model building and evaluation procedures [16], the dOFV diagnostic should be an integral part of model assessment. This is particularly true as the performance of the different methods to obtain estimates of parameter uncertainty remains unclear. The methods themselves are not standardized: bootstrap for example can be done in many different ways [17], both in the computation of the bootstrapped datasets (nonparametric and parametrics methods, stratification strategy) and in the computation of the resulting uncertainty (for example handling of samples for which minimization was not successful, bias-correction [18], Winsorization [19]). Recent work based on extensive simulations [20, 21] has investigated the performance of the covariance matrix and different bootstrap methods and provided some guidance towards in which settings to use which method. However, simulation studies will never be able to cover the full space of possible designs, models and methods. A diagnostic for assessing the appropriateness of a particular method with a particular model and data is thus greatly needed.

### Limitations of the dOFV distribution diagnostic

Two assumptions were made when using the theoretical distribution as reference distribution in the dOFV diagnostic: that the dOFV distribution follows a Chi square distribution at the investigated sample sizes, and that the degrees of freedom of this distribution corresponds to the total number of estimated parameters for NLMEM. These assumptions were tested by computing the SSE dOFV distributions, which correspond to the empirical dOFV distributions obtained by fitting the model on data simulated from that model. In all investigated examples, the SSE dOFV distributions did not differ from the theoretical dOFV distributions and followed a Chi square distribution with degrees of freedom equal to the total number of estimated parameters. This allowed the use of the theoretical distribution in the investigated settings. Generalizing these results, it was expected that the dOFV distribution would follow a Chi square distribution at commonly used sample sizes in NLMEM. However, it remains questionable whether the degrees of freedom would always be equal to the number of estimated parameters, in particular under higher nonlinearity or additional parameter constraints. In any case, the degrees of freedom can only be at or below the number of parameters, so any dOFV distribution above the theoretical distribution is known to be a suboptimal description of parameter uncertainty. If the dOFV distribution of a proposed uncertainty were to be below the theoretical, the authors recommend performing an SSE-type exercise in order to obtain a more precise estimate of the expected dOFV distribution. No formal test such as the Kolmogorov–Smirnoff test was performed to assess whether the dOFV distribution was significantly different than the theoretical distribution. Such a test was not considered for two reasons. First, it was uncertain to which extent the theoretical distribution would correspond to the true empirical distribution for all NLMEM. Second, the degrees of freedom was judged to provide more information on the extent of the inadequacy than a yes/no answer from a formal hypothesis test.

### Bootstrap adequacy in real data examples

In addition to the development of the dOFV diagnostic, this work also provided insight on the performance of case bootstrap in a number of scenarios. Bootstrap proved unsuitable for the real data examples investigated. The estimated degrees of freedom of the dOFV distributions were more than 1.5-fold their expected value. The data contained moderate numbers of individuals (59–74), but few observations per individual as well as unbalanced designs. Regarding the models, their structures were simple (linear processes and well informed covariate relationships) but featured many random effects, especially in the pefloxacin example. It is important to note that no stratification was performed here. Stratification on the number of observations per individual could have been beneficial in both examples, as the number of observations per patient spanned rather heterogeneous ranges (between 1 and 6 for phenobarbital and between 3 and 9 for pefloxacin). However, the number of strata to use would not have been straightforward and would potentially have led to too small subgroups. Stratification on other variables such as covariates included in the model or dose were not considered here as their distribution was homogenous or irrelevant. In the pefloxacin example, 5 % of the bootstrap runs failed and 50 % had one or more unestimable variance parameters, highlighting further limitations of the bootstrap when minimization is problematic. The dOFV distribution using only successful runs without boundary issues differed from the presented distribution, which included all runs (data not shown). Excluding problematic runs lead to a distribution closer to the Chi square distribution, with a degrees of freedom of 14 instead of 16.5. CI were also modified, confirming that the way bootstrap runs are handled can influence the results. Lastly, simulations based on the published models and realized designs were performed to test the influence of model misspecification in the observed inadequacy of the bootstrap: inadequacy was still apparent, but to a lesser extent. These results confirmed that the performed bootstrap was suboptimal, but showed that part of the discrepancy seen with the real data was due to model misspecification.

### Bootstrap adequacy in simulation examples

Compared to the real data examples, data in the simulation examples was richer and more balanced, with the number of individuals varying from 20 to 200. The PK model was similar to the real data example, but the PD model, a sigmoid Emax function, contained more nonlinear parameters. Both examples showed similar increases in degrees of freedom at low sample size as the simulations based on the real data examples, i.e. increases around 25 % over the expected value. The simulation examples enabled to link the global adequacy of the uncertainty estimated by the bootstrap (over all parameters), as measured by changes in dOFV distribution, to the local adequacy of this uncertainty (for each parameter separately). The observed inadequacy of the bootstrap at low sample size could thus be attributed to a suboptimal estimation of the uncertainty of the random effects parameters only, as shown by less datasets than expected including the true simulation value (i.e. suboptimal coverage). Focusing on these parameters, it became apparent that at low sample sizes bootstrap CI around random effects were decreased as well as shifted downwards, underestimating both the actual values of the random effect and their uncertainty, especially in the upper bounds.

### Difficulty of defining sample sizes sufficient for the bootstrap to be adequate

The performance of case bootstrap is expected to depend on the number of “cases”, i.e. individuals in typical NLMEM settings, which is why the simulations investigated the impact of increasing number of individuals. However, the investigated examples highlighted the complexity of defining an appropriate dataset size for bootstrap: similar changes in degrees of freedom were observed for datasets with 20 individuals as for datasets with 60–70 individuals. The performance of bootstrap thus does not only depend on the number of cases. It also depends upon the homogeneity of the information content between the cases, or an appropriate stratification strategy under heterogeneity, so as to preserve the overall information content of the bootstrapped dataset. However, stratification decreases the effective sample size and introduces variability. In addition, the definition of strata may indeed not be straightforward in many clinical settings where design parameters are very different between individuals, and may thus lead to different results under different stratifications. Another issue for mixed models is that the information contained in an individual about a parameter does not only depend on the design, but also on the individual’s parameter value. Stratification at the individual parameter level is however highly problematic. Lastly, the information content of the data always needs to be related to the size and complexity of the model. Typically, richer datasets lead to more complex models and thus the effective information content may actually be similar between datasets of various sizes for models of various sizes. In addition, the different parameters of nonlinear models are not informed equally by a given design, and thus in theory the adequacy of uncertainty estimates should be defined at the parameter level.

### Using parameter-specific “effective” sample sizes to better identify when bootstrap is adequate

Instead of trying to assess the adequacy of bootstrap based on the number of individuals, one could try to quantify the information content for each parameter separately, taking all these factors into account. A possibility for doing so could be to calculate an effective sample size for each parameter. The effective sample size represents how many individuals with perfect information the estimated uncertainty for one parameter corresponds to. Calculating the effective sample size can be done for fixed and random effects using the formulas for standard errors of means (Eq. 4) and variances (Eq. 5). In the simple case of a model where all fixed effects are associated with one random effect, the effective sample size $N$ can then be calculated as follows:

4 $$SE\left( {\bar{X}} \right)\; = \;\frac{SD\left( X \right)}{\sqrt N }\; \to \;N\; = \; \frac{VAR\left( X \right)}{{VAR\left( {\bar{X}} \right)}}$$

where $\bar{X}$ is the estimated fixed effect for the population, $X$ is the vector of the individual parameters, $SD(X)$ is the standard deviation of the individual parameters, $N$ is the effective sample size.$VAR(X)$ corresponds to the variance of the random effect associated with $\bar{X}$ and $SE(\bar{X})$ to the estimate of the standard error of the fixed effect.

5 $$SE\left( {VAR(X)} \right)\; = \;VAR\left( X \right)\sqrt {\frac{2}{N - 1}} \; \to \;N\; = \;2\left( {\frac{VAR\left( X \right)}{{SE\left( {VAR\left( X \right)} \right)}}} \right)^{2} \; + \;1\; = \;2\left( {\frac{1}{{RSE\left( {VAR\left( X \right)} \right)}}} \right)^{2} \; + \;1$$

where $VAR(X)$ is the estimated random effect variance, $SE\left( {VAR(X)} \right)$ its estimated standard error, $N$ is the effective sample size, $SD\left( X \right)$ is the estimated random effect standard deviation and $RSE\left( {VAR\left( X \right)} \right)$ is the relative standard error of $VAR\left( X \right)$.

**Fig. 5:**
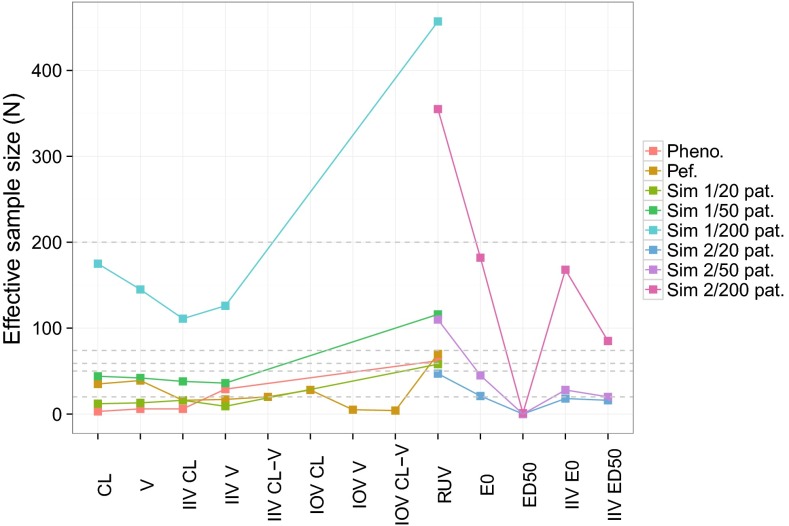
Effective sample sizes calculated for selected model parameters of the real data and simulation examples based on bootstrap uncertainty estimates. *Colors* correspond to the different examples and dataset sizes. *Grey dashed lines* correspond to the total number of individuals in the different examples. *Pheno.* phenobarbital, *Pef.* pefloxacin, *Sim* simulation, *pat.* patients, *CL* clearance, *V* volume of distribution, *IIV* inter-individual variability, *IOV* inter-occasion variability, *RUV* residual unexplained variability, *E0* baseline, *ED50* dose leading to half the maximum effect

The effective sample size for fixed effects and inter-individual variances is expected to be at maximum the total number of individuals in the dataset. For inter-occasion variances, the effective sample size is at maximum the total number of occasions (i.e. the sum of the number of occasions per individual) minus the total number of individuals, as random effects related to inter-individual variances need to be differentiated from those related to inter-occasion variances. Similarly, for residual error variances, $N$ can be as high as the total number of observations minus the number of individuals and minus the sum of the number of occasions per individual. Effective sample sizes were calculated for the real and simulated data examples and are displayed in Fig. 5. For phenobarbital, the number of individuals with perfect information was below 6 for CL, V and IIV CL but at 30 for IIV V. For pefloxacin, the effective sample size was around 30 for fixed effects, between 15 and 20 for inter-individual variances and very low for IOV V. The effective sample size for the RUV was close to the total number of individuals in both real examples. Such low effective sample sizes support the fact that bootstrap was not appropriate in these examples. Effective sample sizes with simulated PK data ranged from 10–15 with 20 patients, to 35–45 with 50 patients and to 110–175 with 200 patients. They were relatively homogenous between parameters. In the PD example, effective sample sizes were lower and more heterogeneous. ED50 presented effective sample sizes close to 0 due to extreme values in the bootstrap driving the standard error at low sample sizes to very high values [SE(ED50) = 10^20^ for 20 patients, 40 000 for 50 patients vs. 1 for 200 patients]. Even with 200 patients the effective sample size was low as the standard error was still much higher than the IIV. For the remaining parameters, effective sample sizes were higher: $N$ was close to the total number of individuals for E0, between half and the total number of individuals for IIV and less than the number of observations minus the number of individuals for RUV. If one wants to establish a cut-off for when the bootstrap is valid, these results could indicate that the minimum number of effective individuals needs to be at least 45, which is the maximum $N$ observed in all examples for which bootstrap was not considered appropriate (real data and simulations below 200 patients). This is a valuable result, however it is important to point out that the concept of effective sample size was developed in an exploratory manner here and thus more investigations are needed if it is to become an established diagnostic. For example, the influence of fixed effects not related to IIV, for which $N$ cannot be calculated, was not investigated. The correlation of $N$ with shrinkage could be of interest in order to use shrinkage as an alternative, potentially more straightforward way to estimate effective sample sizes. A high correlation between these two metrics, with high shrinkage corresponding to low $N$, was observed in the investigated example, but the establishment of a quantitative relationship was not attempted. The impact of covariate effects on the $N$ of corresponding fixed effects may also need to be investigated: for the phenobarbital example, $N$ for CL and V increased 6-fold when fixing the covariate effect instead of estimating it. Lastly, the precision around the calculated $N$ was not quantified in this work. $N$ was observed to be sometimes quite variable between simulated datasets, which may need to be considered if taking decisions based on the calculated effective sample size.

## Conclusion

A diagnostic based on dOFV distributions was developed and is recommended to be routinely used to assess the appropriateness of a given parameter uncertainty distribution. A bootstrap dOFV distribution higher than the theoretical distribution translated into an underestimation of the medians and the CI widths of the random effects. Case bootstrap proved unsuitable for datasets for sample sizes up to 70 individuals, but sample size was not deemed a good predictor of bootstrap appropriateness. Parameter-specific “effective” sample sizes, showing good bootstrap results above 45 effective individuals, could be used instead, but require more investigation.
